# Suppressive Effect of Tetrahydrocurcumin on *Pseudomonas aeruginosa* Lipopolysaccharide-Induced Inflammation by Suppressing JAK/STAT and Nrf2/HO-1 Pathways in Microglial Cells

**DOI:** 10.1155/2022/4978556

**Published:** 2022-03-11

**Authors:** Hui-Wen Lin, Tzu-Chun Chen, Jui-Hsuan Yeh, Shang-Chun Tsou, Inga Wang, Ting-Jing Shen, Chen-Ju Chuang, Yuan-Yen Chang

**Affiliations:** ^1^Department of Optometry, Asia University, Taichung 41354, Taiwan; ^2^Department of Medical Research, China Medical University Hospital, China Medical University, Taichung, Taiwan; ^3^Institute of Medicine, Chung Shan Medical University, Taichung 40201, Taiwan; ^4^Department of Nutrition, Chung Shan Medical University, Taichung 40201, Taiwan; ^5^Rehabilitation Sciences & Technology, University of Wisconsin-Milwaukee, Milwaukee, WI, USA; ^6^Department of Microbiology and Immunology, School of Medicine, Chung-Shan Medical University, Taichung 40201, Taiwan; ^7^Emergency Department, Kaohsiung Municipal United Hospital, Kaohsiung 80457, Taiwan; ^8^Clinical Laboratory, Chung Shan Medical University Hospital, Taichung 40201, Taiwan

## Abstract

Brain inflammation, a pathological feature of neurodegenerative disorders, exhibits elevated microglial activity and increased levels of inflammatory factors. The present study was aimed at assessing the anti-inflammatory response of tetrahydrocurcumin (THC), the primary hydrogenated metabolite of curcumin, which was applied to treat *Pseudomonas aeruginosa* (*P.a.*) lipopolysaccharide- (LPS-) stimulated BV2 microglial cells. THC reduced *P.a.* LPS–induced mortality and the production of inflammatory mediators IL-6, TNF-*α*, MIP-2, IP-10, and nitrite. A further investigation revealed that THC decreased these inflammatory cytokines synergistically with JAK/STAT signaling inhibitors. THC also increased Nrf2/HO-1 signaling transduction which inhibits iNOS/COX-2/pNF*κ*B cascades. Additionally, the presence of the HO-1 inhibitor Snpp increased the levels of IP-10, IL-6, and nitrite while THC treatment reduced those inflammatory factors in *P.a.* LPS–stimulated BV2 cells. In summary, we demonstrated that THC exhibits anti-inflammatory activities in *P.a.* LPS-induced inflammation in brain microglial cells by inhibiting STAT1/3-dependent NF-*κ*B activation and inducing Nrf2-mediated HO-1 expression.

## 1. Introduction

Neurodegenerative disorders such as Alzheimer's disease (AD), Parkinson's disease, amyotrophic lateral sclerosis, and frontotemporal dementia exhibit brain inflammation [1, 2]. Many factors can induce central nervous system (CNS) inflammation, including immune system dysregulation, bacterial infection, viral infection, and parasite invasion [3]. Lipopolysaccharide (LPS), the major component of the outer membrane of Gram-negative bacteria, is the most potent microbial inducer of inflammation and sepsis. LPS is reported to be an immunostimulatory ligand for toll-like receptor 4 (TLR-4), which is primarily expressed in brain resident macrophages and microglia in the CNS [4]. Upon inflammation, microglial cells are activated and release proinflammatory factors, such as tumor-necrosis factor-*α* (TNF-*α*), interleukin- (IL-) 6, and IL-1*β*. Moreover, activated microglia trigger the production of reactive oxygen species (ROS) and nitric oxide (NO) which cause damage in axons and neurons [5, 6]. Studies have reported that stimulation of LPS (from *Escherichia coli*) increases the expression of TLR-4, TNF-*α*, IL-6, cyclooxygenase-2 (COX-2), NO, and phosphonuclear factor kappa B (pNF*κ*B) in microglial BV2 cells [7, 8]. Furthermore, the anti-inflammation property of different inflammation signal pathways has yet to be demonstrated. In this study, we used microglial BV2 cells as an *in vitro* model of *Pseudomonas aeruginosa* (*P.a.*) LPS–induced inflammation.

Tetrahydrocurcumin (1,7-bis (4-hydroxy-3-methoxyphenyl) heptane-3,5-dione, C_21_H_24_O_6_, abbreviated as THC) is the primary hydrogenated metabolite of curcumin (*Curcuma longa* Linn); it also functions as an antihypertensive, antidiabetic, antioxidant, anti-inflammatory, and anticancer agent [9, 10]. In a rat model of AD, THC reduces ROS levels and protects cells from amyloid *β*- (A*β*-) induced cytotoxicity [11]. THC has also been reported to reduce the severity of pathological defects of AD by inhibiting cell-cycle arrest and apoptosis of amyloid *β*-treated BV2 cells through the regulation of Ras-extracellular signal-regulated kinase signaling [12]. Additionally, in a paw edema mouse model, THC inhibits the COX-2-NF*κ*B pathway by inactivating transforming growth factor, which eventually reduces inflammation [13]. In LPS-stimulated RAW264.7 cells, THC exerts potent anti-inflammatory and antioxidant activities through the inhibition of the generation of ROS, NO, and monocyte chemotactic protein-1 [14]. Many previous studies have indicated that inhibition of the anus kinase- (JAK-) signal transducer and activator of transcription protein (STAT) signaling pathway can reduce LPS-induced inflammation [15–17]. A recent study reported that in LPS-stimulated macrophages, inducing the expression of HO-1 can inhibit the expression of inflammatory cytokines and thereby inhibit the M1 polarization of macrophages [15]. Moreover, over the years, an increasing number of therapeutic agents have been developed that exert their antioxidant and anti-inflammatory effects by inducing HO-1 expression. As far as we know, there is no evidence regarding the effects of THC in *P.a.* LPS–induced brain inflammation. Furthermore, there is no published report about THC inhibiting *P.a.* LPS–induced inflammation response through JAK/STAT and Nrf2/HO-1 signaling pathways in BV2 microglial cells. This study demonstrates the possible mechanisms by which THC acts against the proinflammatory mediators produced by *P.a.* LPS–induced BV2 microglial cells.

## 2. Materials and Methods

### 2.1. Cells

The murine BV2 microglial cell line was obtained from Dr. Dah-Yuu Lu (China Medical University, Taichung, Taiwan). Cells were maintained in Dulbecco's modified Eagle's medium containing 10% heat-inactivated fetal bovine serum (Cat#10437, Gibco), and 100 ng/ml penicillin/streptomycin (Cat#15140, Gibco) at 37°C in a humidified incubator containing 5% CO_2_.

### 2.2. Agents and Antibodies

Tetrahydrocurcumin (THC, C_21_H_24_O_6_) was dissolved in DMSO and stored (Cat#sc-391609, Santa Cruz, CA, USA). *P. a.* LPS was prepared with PBS and stored (Cat#L9143, Sigma-Aldrich). STAT3 inhibitor AG490 was purchased from Cayman Chemical. JAK inhibitor WP1066 was purchased from Calbiochem. Cell Counting Kit-8 (CCK-8) was purchased from Dojindo Molecular Technologies, Inc. (Rockville, MD, USA). Antibodies against p-JAK2, p-STAT1, p-NF*κ*B p50, and HO-1 inhibitor tin protoporphyrin IX (Snpp) were purchased from Santa Cruz Biotechnology Inc. Antibodies against p-STAT3, iNOS, COX2, and HO-1 were purchased from Abcam. Antibody against p-Nrf2 was purchased from Invitrogen. Antibody against glyceraldehyde 3-phosphate dehydrogenase (GAPDH) was purchased from Taiclone.

### 2.3. Western Blotting

Western blotting was performed according to our previous study [16]. Briefly, cells were harvested at indicated time points then extracted using a lysis buffer containing protease inhibitors (Sigma-Aldrich) on ice for 15 min. Subsequently, the samples were added with protein dye and were heated to 100°C for 15 min. After samples were cooled down on ice, sodium dodecyl sulfate polyacrylamide (SDS) gel electrophoresis was applied to separate the proteins. Then, proteins were transferred to a polyvinylidene difluoride (PVDF) membrane (Millipore). Next, the membrane was blocked with 5% nonfat milk in phosphate-buffered saline containing 0.05% Tween-20 (PBS-T) at room temperature for 1 h. Membrane was washed 3 times with PBS-T, and the primary antibodies were used to immunohybridize the indicated proteins at 4°C overnight. Protein-antibody complexes were then incubated with the indicated horseradish peroxidase- (HRP-) conjugated secondary antibodies at room temperature for 1 h. Afterward, by using an enhanced chemiluminescence western blot detection kit, the protein–antibody complexes with HRP on the PVDF membrane were detected and the signals were captured with an image system.

### 2.4. Enzyme-Linked Immunosorbent Assay (ELISA)

At indicated time points, the cell culture supernatants were collected and stored at -80°C. The concentrations of mouse IL-6 (Cat#431316, BioLegend), TNF-*α* (Cat#430916, BioLegend), MIP-2 (Cat#ab211762, Abcam), and IP-10 (Cat#ab275364, Abcam) in the samples were determined by using ELISA kits following the manufacturer's instructions. The absorbance of the immunocomplex was determined at 450/595 nm using ELISA reader (Multiskan Spectrum, Thermo Co., Vantaa, Finland).

### 2.5. Nitrite Detection Assay

The concentration of nitrite in the medium was determined as the indicator of NO production according to previously described methods [16]. Briefly, 150 *μ*l of sampled supernatant was mixed with 100 *μ*l of Griess reagent (Cat#G4410, Sigma-Aldrich) and incubated for 10 min at room temperature. The absorbance of the mixture was determined at 595 nm using ELISA reader (Multiskan Spectrum, Thermo Co., Vantaa, Finland).

### 2.6. Statistical Analysis

One-way ANOVA (Tukey's multiple comparison test) was used to analyzed the data and compare the investigated groups. The statistical findings were expressed as mean ± standard deviation (SD). All *p* values were obtained by performing two-tailed significance tests. Bars with the same letter represent no significant difference between the groups. Bars with different letters indicate a statistically significant (*p* < 0.05) difference between the groups.

## 3. Results

### 3.1. THC Reduces P. aeruginosa LPS–Induced Inflammation

**Figure 1:**
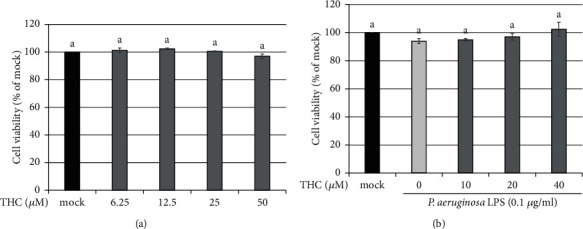
THC treatment exerts no significant effect on cell viability of BV2 microglial cells with or without *P. a.* LPS stimulation. (a) BV2 microglial cells were untreated (the mock group) or pretreated with THC (6.25, 12.5, 25, or 50 *μ*M) for 24 h. (b) BV2 microglial cells were untreated (the mock group) or pretreated with THC (10, 20, or 40 *μ*M) for 1.5 h following stimulation with *P. a.* LPS (0.1 *μ*g/ml) for 24 h. CCK analysis indicated cell viability. The experimental quantitative data are presented in terms of the mean ± SD (*n* = 3). The treatment and mock group significantly differed. Bars with the same letter indicate no significant difference between the groups.

CNS inflammation, a key event in the pathogenesis and progression of neurodegenerative diseases, is mediated by activated microglial cells [4]. To investigate the potential anti-inflammatory activity of THC in the brain, we treated murine BV2 microglial cells with THC. Cell Counting Kit-8 (CCK-8) assays verified that various doses (6.25, 12.5, 25, and 50 *μ*M) of THC had no significant effect on the cell viability of BV2 microglial cells (Figure 1(a)). Under *P.a.* LPS (0.1 *μ*g/ml) stimulation, the cell viability of cells pretreated with various doses (10, 20, and 40 *μ*M) of THC was comparable (Figure 1(b)).

**Figure 2:**
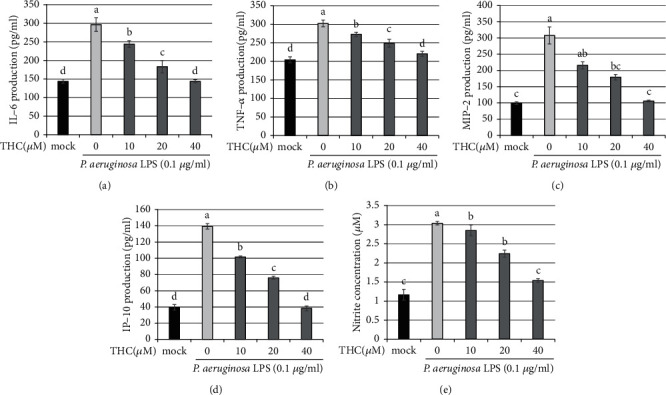
THC reduces the production of IL-6, TNF-*α*, MIP-2, IP-10, and nitrite in *P. a.* LPS–stimulated BV2 microglial cells. BV2 microglial cells were untreated (the mock group) or pretreated with THC (10, 20, or 40 *μ*M) for 1.5 h before being stimulated by *P. a.* LPS (0.1 *μ*g/ml) for 24 h. The production of (a) IL-6, (b) TNF-*α*, (c) MIP-2, (d) IP-10, and (e) nitrite was detected using ELISA. The experimental quantitative data are presented in terms of the mean ± SD (*n* = 3). The mock group was considered as a control group. Bars with the same letter represent no significant difference between the groups. Bars with different letters indicate a statistically significant (*p* < 0.05) difference between the groups.

Subsequently, the BV2 microglial cells were pretreated with THC (10, 20, or 40 *μ*M) for 1.5 h and then stimulated with *P.a.* LPS (0.1 *μ*g/ml) for 24 h. Results of ELISA indicated that the production of inflammatory cytokines and chemokines—including IL-6 (Figure 2(a)), TNF-*α* (Figure 2(b)), MIP-2 (Figure 2(c)), IP-10 (Figure 2(d)), and nitrite (Figure 2(e))—was significantly increased by *P.a.* LPS stimulation relative to the mock (control) samples. However, THC pretreatment notably reduced these inflammatory factors in a dose-dependent manner. Thus, THC exhibits anti-inflammatory capacity without cytotoxicity.

### 3.2. THC Decreases Inflammation through Inhibiting JAK-STAT

**Figure 3:**
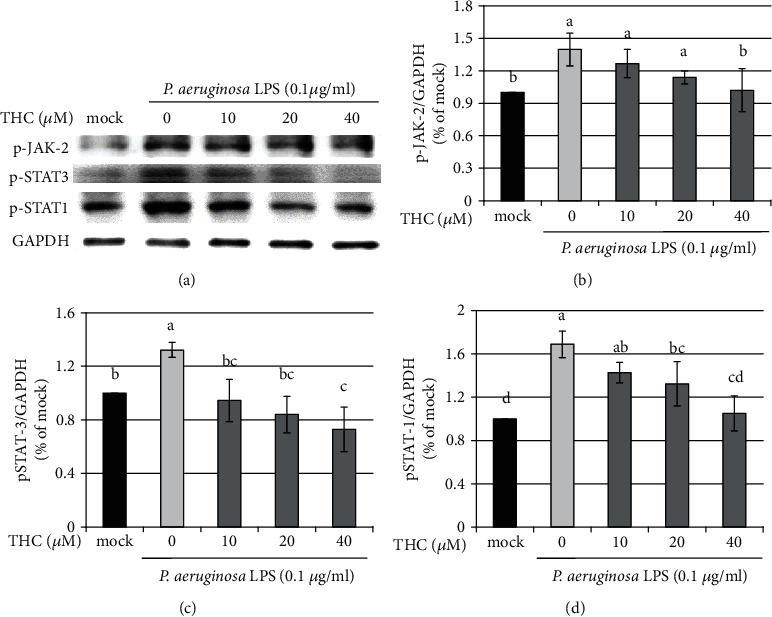
THC attenuates p-JAK-2, p-STAT3, and p-STAT1 protein expressions in *P. a.* LPS–stimulated BV2 microglial cells. BV2 microglial cells were untreated (the mock group) or pretreated with THC (10, 20, or 40 *μ*M) for 1.5 h before being stimulated by *P. a.* LPS (0.1 *μ*g/ml) for 24 h. (a) Western blotting was used to obtain the expressions of p-JAK2, p-STAT3, p-STAT1, and GAPDH proteins. Statistical results of (b) p-JAK2, (c) p-STAT3, and (d) p-STAT1 were shown. The mock group was considered as the control group. The experimental quantitative data are presented in terms of the mean ± SD (*n* = 3). Bars with the same letter represent no significant difference between the groups. Bars with different letters indicate a statistically significant (*p* < 0.05) difference between the groups.

JAK-STAT signaling transduction has been reported to be a crucial inflammatory pathway [17, 18]. We pretreated BV2 microglial cells with THC (10, 20, or 40 *μ*M) for 1.5 h and then stimulated them with *P.a.* LPS (0.1 *μ*g/ml) for 24 h. Western blotting was used to identify the protein expression (Figure 3(a)), and the statistical results of p-JAK2 (Figure 3(b)), p-STAT3 (Figure 3(c)), and p-STAT1 (Figure 3(d)) were shown. The expressions of these proteins were significantly increased by *P.a.* LPS stimulation and were also reduced by THC pretreatment. Furthermore, these inhibitory effects were greater at larger dose concentrations of THC.

**Figure 4:**
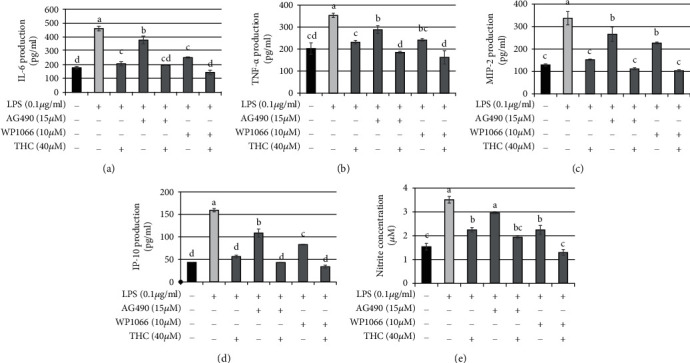
STAT3/JAK blocker combined with THC reduces IL-6, TNF-*α*, MIP-2, IP-10, and nitrite production in *P. a.* LPS–stimulated BV2 microglial cells. BV2 microglial cells were pretreated with STAT3 inhibitor AG490 (15 *μ*M), JAK inhibitor WP1066 (10 *μ*M), or THC (40 *μ*M) for 1.5 h before being stimulated by *P. a.* LPS (0.1 *μ*g/ml) for 24 h. ELISA was used to detect the production of (a) IL-6, (b) TNF-*α*, (c) MIP-2, (d) IP-10, and (e) nitrite from BV2 microglial cells under *P. a.* LPS stimulation. The experimental quantitative data are presented in terms of the mean ± SD (*n* = 3). Bars with the same letter represent no significant difference between the groups. Bars with different letters indicate a statistically significant (*p* < 0.05) difference between the groups.

To further examine the role of JAK-STAT signaling, we pretreated BV2 microglial cells with STAT3 inhibitor AG490 (15 *μ*M), JAK inhibitor WP1066 (10 *μ*M), or THC (40 *μ*M) for 1.5 h following stimulation with *P.a.* LPS (0.1 *μ*g/ml) for 24 h. ELISA analysis results indicated that the production of IL-6 (Figure 4(a)), TNF-*α* (Figure 4(b)), MIP-2 (Figure 4(c)), IP-10 (Figure 4(d)), and nitrite (Figure 4(e)) was increased in cells stimulated with *P.a.* LPS but decreased in cells pretreated with STAT3 inhibitor AG490 or JAK inhibitor WP1066. Moreover, THC pretreatment and the combination of THC with JAK-STAT inhibitors indicated the strong inhibition activity of *P.a.* LPS–exposed cells. Thus, THC reduces inflammation by targeting JAK-STAT signaling.

### 3.3. THC Reduces Oxidative iNOS-COX-2-p-NF*κ*B Signaling

**Figure 5:**
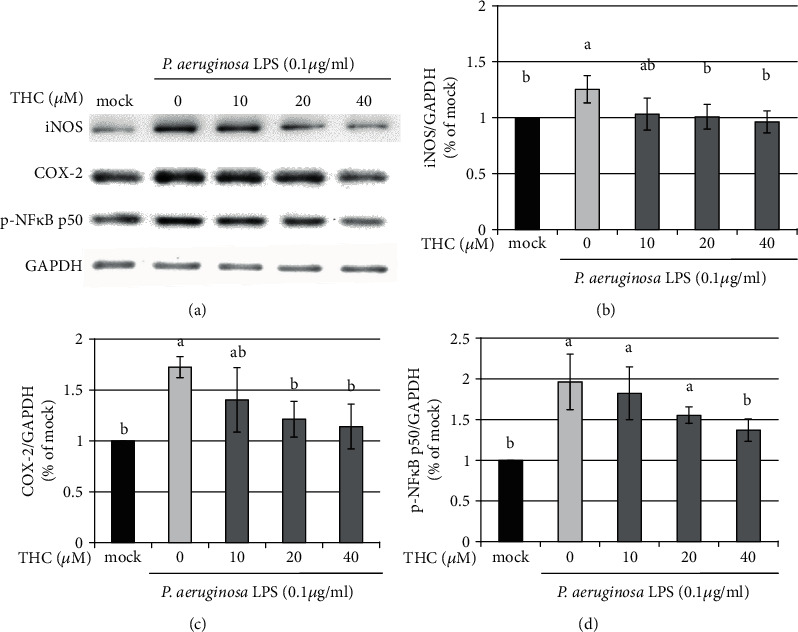
THC inhibits iNOS, COX-2, and p-NF*κ*B expressions in *P. a.* LPS-stimulated BV2 microglial cells. (a) BV2 microglial cells were pretreated without (mock) or with THC (10, 20, or 40 *μ*M) for 1.5 h following stimulation with *P. a.* LPS (0.1 *μ*g/ml) for 24 h. Western blotting showed the protein expression of iNOS, COX-2, p-NF*κ*B, and GAPDH. Statistical results of (b) iNOS, (c) COX-2, and (d) p-NF*κ*B p50 were shown. The experimental quantitative data are presented as means ± SD (*n* = 3). Bars with the same letter represent no significant difference between the groups. Bars with different letters indicate a statistically significant (*p* < 0.05) difference between the groups.

It is reported that LPS stimulation increases the expressions of TLR-4, TNF-*α*, IL-6, COX-2, iNOS, and p-NF*κ*B in microglial BV2 cells [19]. In the present study, BV2 microglial cells were pretreated with THC (10, 20, or 40 *μ*M) for 1.5 h and then stimulated with *P.a.* LPS (0.1 *μ*g/ml) for 24 h. Western blotting was used to determine protein expression (Figure 5(a)), and the statistical analysis of iNOS (Figure 5(b)), COX-2 (Figure 5(c)), and p-NF*κ*B (Figure 5(d)) was shown. *P.a.* LPS stimulation significantly induced the expression of these proteins; however, THC reduced the expression of iNOS, COX-2, and p-NF*κ*B proteins in a dose-dependent manner. Therefore, THC inhibits oxidative iNOS-COX-2-p-NF*κ*B signaling transduction.

### 3.4. THC Defenses against Inflammation through Activation of Nrf2-HO-1 Signaling

**Figure 6:**
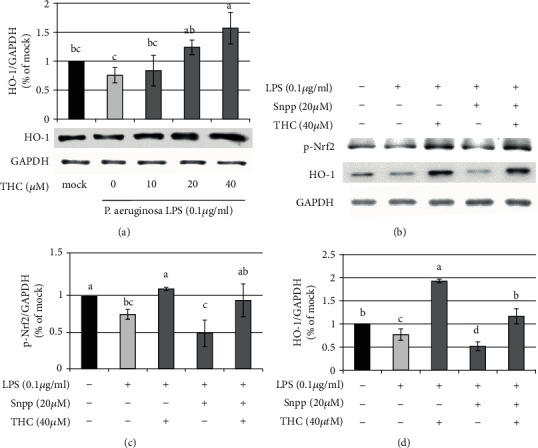
THC reverses the inhibition of HO-1 inhibitor Snpp in *P. a.* LPS–stimulated BV2 microglial cells. (a) BV2 microglial cells were untreated (the mock group) or pretreated with THC (10, 20, or 40 *μ*M) for 1.5 h before being stimulated with *P. a.* LPS (0.1 *μ*g/ml) for 24 h. Western blotting was used to obtain the expressions of p-Nrf2, HO-1, and GAPDH proteins. (b) BV2 microglial cells were pretreated with THC (40 *μ*M) or HO-1 inhibitor Snpp (20 *μ*M) for 1.5 h before being stimulated with *P. a.* LPS (0.1 *μ*g/ml) for 24 h. Western blotting was used to obtain the expressions of p-Nrf2, HO-1, and GAPDH proteins. Statistical results of (c) p-Nrf2 and (d) HO-1 were shown. The experimental quantitative data are presented in terms of the mean ± SD (*n* = 3). Bars with the same letter represent no significant difference between the groups. Bars with different letters indicate statistical significance (*p* < 0.05) between the groups.

HO-1 functions not only to reduce oxidative injury but also to regulate inflammatory responses [20]. Additionally, HO-1 interacted with Nrf2 to defend against oxidative stress damage [21, 22]. We pretreated BV2 microglial cells with THC (10, 20, or 40 *μ*M) for 1.5 h prior to stimulation with *P.a.* LPS (0.1 *μ*g/ml) for 24 h. Western blotting indicated that the expression of the HO-1 protein was induced by THC administration in a dose-dependent manner (Figure 6(a)). Notably, the expressions of p-Nrf2 (Figures 6(b) and 6(c)) and HO-1 (Figures 6(b) and 6(d)) were reduced by the HO-1 inhibitor Snpp (20 *μ*M) but increased by THC (40 *μ*M) treatment.

**Figure 7:**
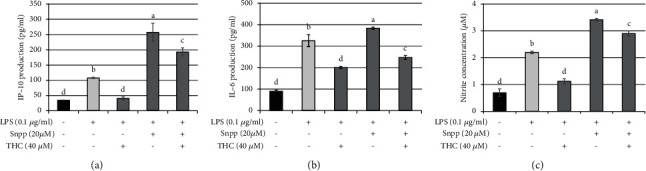
THC treatment reduces Snpp-prompted IP-10, IL-6, and nitrite production in *P. a.* LPS–stimulated BV2 microglial cells. BV2 microglial cells were pretreated with THC (40 *μ*M) or HO-1 inhibitor Snpp (20 *μ*M) for 1.5 h before being stimulated with *P. aeruginosa* LPS (0.1 *μ*g/ml) for 24 h. The production of (a) IP-10, (b) IL-6, and (c) nitrite was determined using ELISA. The experimental quantitative data are presented in terms of mean ± SD (*n* = 3). Bars with the same letter represent no significant difference between the groups. Bars with different letters indicate a statistically significant (*p* < 0.05) difference between the groups.

Subsequently, we detected the presence of inflammatory factors using ELISA. The production of IP-10 (Figure 7(a)), IL-6 (Figure 7(b)), and nitrite (Figure 7(c)) was increased in cells stimulated with *P.a.* LPS (0.1 *μ*g/ml). The application of HO-1 inhibitor Snpp (20 *μ*M) further exacerbated the inflammatory response. However, THC treatment reduced the production of these inflammatory factors, indicating that THC played a crucial role in the anti-inflammatory response. Therefore, these results indicate that THC drives Nrf2-HO-1 signaling to reduce inflammation.

## 4. Discussion

**Figure 8:**
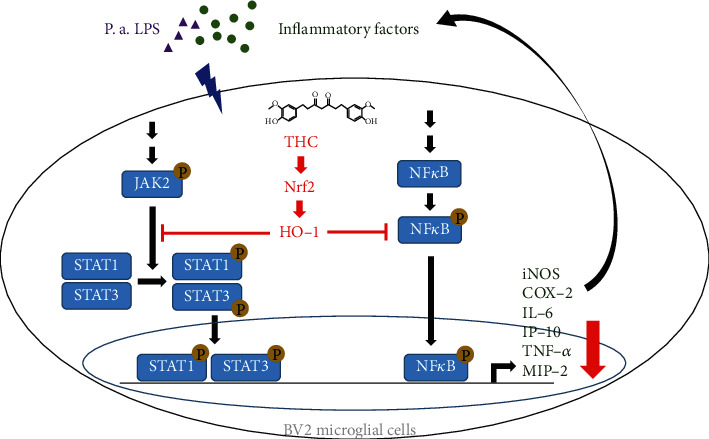
Conclusion of this study. THC blocks *P. a.* LPS–induced oxidative responses by increasing Nrf2-HO-1 expression which attenuates the iNOS, COX-2, and p-NF*κ*B expression. THC also inhibits the level of *P. a.* LPS–prompted JAK-STAT signaling and the inflammatory mediators IL-6, TNF-*α*, MIP-2, and IP-10 productions. Collectively, THC is a potent anti-inflammatory agent in brain encephalitis.

The activation of microglial cells and the immune response are essential for defending against the invasion of pathogens and many other stimuli, such as the Gram-negative bacterial wall component LPS. However, an excessive microglia-mediated inflammatory response may also result in brain damage. In this study, by applying THC, the primary hydrogenated metabolite of curcumin, to *P.a.* LPS–stimulated microglia BV2 cells, we observed that THC suppressed the levels of inflammatory mediators, including IL-6, TNF-*α*, MIP-2, IP-10, and nitrite. Additionally, THC downregulated the JAK/STAT inflammatory pathway. In addition to exhibiting anti-inflammatory activity, THC also exhibited antioxidative effects due to its ability to inhibit the iNOS/COX2/NF*κ*B signaling cascade. By contrast, the expressions of HO-1 and p-Nrf2 were enhanced. These factors jointly indicate that THC is a neuroprotective agent for encephalitis induced by LPS treatment (Figure 8).

Curcumin (1,7-bis (4-hydroxy-3-methoxyphenyl)-1,6-heptadiene-3,5-dione) is a common natural flavouring substance that is extensively used in various foods. Furthermore, accumulating evidence has indicated that curcumin offers multiple health benefits, such as reducing the incidence of metabolic syndrome, pain, and degenerative eye diseases by targeting multiple inflammatory and oxidative signaling molecules [23–25]. However, the low absorption and rapid metabolism of curcumin result in its poor systemic bioavailability. The major metabolite of curcumin, THC, is reported to exhibit stable activity and absorption efficiency and to possess a more favourable bioavailability than that of curcumin [10, 26, 27]. Xie et al. have demonstrated that THC exerts more notable anti-inflammatory and antioxidant activities in LPS-stimulated RAW264.7 macrophages than curcumin does [14]. Moreover, compared with curcumin, THC more effectively suppresses pathways for COX-2 and transforming growth factor *β*, activated kinase-1, and NF-*κ*B *in vivo* [13]. We therefore applied THC in our study and demonstrated that THC is a strong anti-inflammatory agent that substantially inhibits inflammatory signaling cascades, cytokines, and chemokines and inhibits oxidative stress.

Neurodegenerative disorders are commonly associated with oxidative stress-induced inflammation [28, 29]. Under LPS stimulation, the TLR4-mediated NF-*κ*B and mitogen-activated protein kinases are activated and iNOS-mediated proinflammatory NO is produced in activated macrophages [14]. LPS is also known to induce COX-2 production *in vitro* and *in vivo* [30–32]. Therefore, targeting oxidative stress is crucial for reducing brain inflammation. Nrf2 is a key factor in protecting cells from oxidative stress and inflammation-induced damage, which modulates the levels of antioxidants and detoxification enzymes, such as HO-1, superoxide dismutase 1, NAD(P)H dehydrogenase 1, and glutathione peroxidase 1 [33]. A recent study has shown that a dried ripe seed of Trichosanthes kirilowii Maximowicz, Trichosanthis semen, could inhibit LPS-induced inflammation in BV2 microglial cells through activating HO-1 and inhibiting NF-*κ*B signaling [34]. Our findings reveal that THC treatment reduces nitrite production (Figure 2(e)). Furthermore, THC not only inhibited iNOS/COX-2/p-NF*κ*B signaling (Figure 5) but also upregulated Nrf2/HO-1 pathways (Figure 6). These results demonstrate the beneficial effects of THC on reducing LPS-induced oxidative injury by increasing the expression of antioxidative factors HO-1 and p-Nrf2. We thus propose that THC can considerably reduce CNS inflammation due to its anti-inflammatory and antioxidative properties.

## 5. Conclusions

To our knowledge, this is the first report implicating the inhibition of *P. a.* LPS-induced inflammatory molecule gene expression by THC in BV2 microglial cells. Our findings demonstrate that THC has excellent anti-inflammatory activities by suppressing the STAT1/3-dependent NF-*κ*B pathway and inducing Nrf2-mediated HO-1 expression. Thus, THC is proposed as a powerful therapeutic agent to treat encephalitis.

## Data Availability

The data that support the findings of this study are available from the corresponding author upon reasonable request.
